# *Wolbachia*: A Bug’s Life in Another Bug

**DOI:** 10.3201/eid1408.080497

**Published:** 2008-08

**Authors:** Didier Raoult

**Affiliations:** * World Health Organization Collaborative Center for Rickettsioses and Other Arthropod Borne Bacterial Diseases, Marseille, France

*Wolbachia*: A Bug’s Life in Another Bug is timely and useful for understanding this bacterial genus. This book covers all aspects of *Wolbachia* organisms from basic science and history to medicine and veterinary science. The authors are the most renowned in their field.

These bacteria live intracellularly (similar to *Rickettsia* spp.) in arthropods or helminths and manipulate their hosts and progeny. They can select specific partners for their infected hosts and favor parthenogenesis or in ovo conversion from male to female. These bacteria were identified in 1924 in arthropods and are a paradigm of host–bacteria relationships. *Wolbachia* organisms became noteworthy in tropical medicine when they were found in nematode worms, specifically those causing filariasis. *Wolbachia* bacteria manipulate the fertility of most filarial worms; treating filariasis patients with antimicrobial drugs results in elimination of *Wolbachia* organisms and the microfilaremia. Unexpectedly, doxycycline was also found to kill the worm. Doxycycline could now be paradoxically recommended to treat certain cases of filariases.

I highly recommend reading this book because I believe that *Wolbachia* science will grow dramatically in the coming years as an example of the complex relationships between intracellular bacteria and hosts. Unfortunately, the most surprising discovery about these bacteria is too recent to be included in this book, namely, that they can integrate nearly their entire genome into the chromosome of their host, a unique example of massive lateral gene transfer. This characteristic may help investigators understand integration of mitochondrial genes into the eukaryotic nucleus.

